# *In Vitro* Analysis of an *Alkalihalobacillus clausii* Spore-Based Probiotic Formulation Clarifies the Mechanisms Underlying Its Beneficial Properties

**DOI:** 10.3390/biom15091294

**Published:** 2025-09-08

**Authors:** Diletta Mazzantini, Marco Calvigioni, Francesco Celandroni, Alessandro Saba, Emilia Ghelardi

**Affiliations:** 1 Department of Translational Research and New Technologies in Medicine and Surgery, University of Pisa, 56123 Pisa, Italy; diletta.mazzantini@unipi.it (D.M.); marco.calvigioni@med.unipi.it (M.C.);; 2 Department of Surgical, Medical, Molecular and Critical Area Pathology, University of Pisa, 56126 Pisa, Italy; 3 Research Center Nutraceuticals and Food for Health—Nutrafood, University of Pisa, 56124 Pisa, Italy

**Keywords:** *A. clausii*, probiotics, spores, β-galactosidase, vitamins, D-lactate, short-chain fatty acids

## Abstract

Probiotics are microorganisms with recognized beneficial properties that are used to improve host health. In particular, probiotics administered as spores, such as those belonging to the genera *Bacillus* and *Alkalihalobacillus*, are attracting great interest due to their high tolerance to gastrointestinal conditions. This *in vitro* study aimed to assess the probiotic attributes potentially contributing to the *in vivo* beneficial effects of a commercial spore-based probiotic formulation composed of four *Alkalihalobacillus clausii* strains. The tolerance and survival of the spores from the formulation in simulated gastrointestinal fluids, as well as their germination rate and adhesion to mucins, were analyzed. Furthermore, metabolic properties of spore-derived vegetative cells were assessed, including lactose degradation and biosynthesis of antioxidant enzymes (catalase and superoxide dismutase), group B vitamins (B_2_, B_8_, B_9_, and B_12_), short-chain fatty acids (acetate, propionate, and butyrate), and D-lactate. *A. clausii* spores were shown to survive in artificial gastric juice, adhere to mucins and germinate *in vitro*, and replicate in simulated intestinal fluid, suggesting their potential resilience in the gastrointestinal tract, where they can exert beneficial effects after germination. *A. clausii* was also able to produce beneficial enzymes and metabolites, including β-galactosidase, catalase, superoxide dismutase, group B vitamins, and short-chain fatty acids, but it was unable to produce D-lactic acid. Our findings highlight the probiotic properties and potential of such *A. clausii* strains in both their spore and vegetative forms, reinforcing the clinical relevance of this multi-strain spore-based formulation for enhancing intestinal health.

## 1. Introduction

Probiotics are defined as “live microorganisms that, when administered in adequate amounts, confer a health benefit on the host” [1]. Beneficial effects of probiotic administration depend on several mechanisms, including gut barrier function amelioration and host immune response enhancement, oxidative stress reduction, competition with pathogens for nutrients and mucosal binding sites, and production of antimicrobial molecules, vitamins, and food-degrading enzymes [2,3,4,5,6]. A plethora of probiotic formulations are available in the global market and find applications in a wide range of clinical conditions [7,8]. *Bifidobacterium*, *Lactobacillus*, *Enterococcus*, *Lactococcus*, and *Streptococcus* strains have been traditionally used as probiotics [4]. Furthermore, probiotic species belonging to the genus *Bacillus* (i.e., *Bacillus subtilis*) or related genera, such as *Alkalihalobacillus* (i.e., *Alkalihalobacillus clausii,* formerly *Bacillus clausii*) and *Heyndrickxia* (i.e., *Heyndrickxia coagulans*, formerly *Bacillus coagulans*), are commonly used [9].

The peculiarity of microorganisms belonging to *Bacillus* and related genera resides in their ability to exist in two distinct states—dormant spores and active vegetative cells—which differ in structure, metabolism, and survival in adverse conditions [10]. Vegetative cells are susceptible to extreme environmental conditions (i.e., temperature, radiation, and pH), including the harsh conditions of the human gastrointestinal tract. Since orally administered probiotics should transit unaffected through the upper digestive tract to exert their beneficial effects in the gut [8], only a tiny fraction of probiotics administered as vegetative cells may survive the transit through the stomach and reach the intestine. Thus, non-spore-forming probiotics are usually administered in capsules or protective matrices to resist the low pH of the stomach. By contrast, spores are naturally able to withstand the challenging conditions of the gastrointestinal tract due to their intrinsic resistance. Upon reaching the intestine, spores can germinate and produce vegetative cells able to proliferate, exert beneficial effects, and, eventually, re-sporulate [11,12,13]. In addition, since spores retain viability during distribution and storage, spore-based probiotics have long shelf lives [11]. For these reasons, most *Bacillus*-based probiotic formulations are commercialized as spores.

Formulations containing spores of one or more *A. clausii* strains are commercialized worldwide, and their beneficial effects in different disorders (i.e., diarrhea, irritable bowel syndrome, infections, and allergy) were highlighted in clinical studies [14,15,16,17,18,19,20,21,22,23,24,25,26,27,28,29,30,31,32]. Some preclinical reports also investigated the beneficial properties and potential mechanisms of action of probiotic *A. clausii* [28,33,34,35,36,37]. In these studies, *A. clausii* has been shown to improve gut functionality and integrity by stimulating the production of mucins and tight junctions, exerting immunomodulatory and antioxidant activities, and producing antimicrobial compounds that could help in counteracting intestinal infections [28,36,38]. However, most of these studies were performed on vegetative cells of strains isolated from commercial formulations [33,35,38,39,40]. The attributes of whole formulations have rarely been evaluated.

In this study, we focused on the drug Enterogermina^®^ (Opella Healthcare, Milan, Italy), a probiotic formulation constituted by spores of four *A. clausii* strains (OC, NR, SIN, and T). In particular, we analyzed the whole product for some selected probiotic properties to clarify some of the mechanisms responsible for the beneficial effects exhibited by the formulation *in vivo*.

## 2. Materials and Methods

### 2.1. Product, Strains, and Culture Conditions

Enterogermina^®^ vials (batch number 2706), containing 2 billion spores of *A. clausii* in 5 mL of water for unit dose, were purchased from local pharmacies and analyzed before expiry. When required, a Brain–Heart Infusion (BHI; Thermo Fisher Scientific, Waltham, MA, USA) medium supplemented with 1% glucose (BHIG), a Luria–Bertani medium (LB; LAB Logistics Group GMbH, Meckenheim, Germany), or trypticase soy agar containing 5% horse blood (TSH; bioMérieux, Marcy-l’Etoile, France) was used.

### 2.2. Quantification of the Microorganisms Contained in the Probiotic Product

The quantification of spores and viable bacteria contained in the formulation was conducted using the plate count method. The content of product vials was transferred to sterile tubes and centrifuged at 4000× *g* for 10 min at 4 °C. Supernatants were discarded, and pellets were suspended in 5 mL of sterile phosphate-buffered saline (PBS; 1 M KH_2_PO_4_, 1 M K_2_HPO_4_, 5 M NaCl, pH 7.2). Aliquots were withdrawn, and half of them were thermally treated at 80 °C for 15 min for spore count. Thermally treated and untreated aliquots were serially diluted in PBS, and 100 μL aliquots were plated on TSH agar plates. Plates were incubated at 37 °C for 48 h, and the number of colony-forming units (CFUs) was determined at the end of the incubation.

### 2.3. Viability of Spores and Growth Evaluation in Simulated Gastrointestinal Fluids

To collect spores, the vial contents were transferred to sterile tubes and centrifuged at 4000× *g* for 15 min at 4 °C, and supernatants were discarded. Spores were directly suspended in 5 mL of simulated gastric fluid containing 0.03 M NaCl, 0.084 M HCl, and 0.32% pepsin in sterile water (pH 1.5; USP Reference Standards, Frederick, MD, USA) and artificial intestinal juice containing 0.3% Oxgall bile salts (Merck KGaA, Darmstadt, Germany) and 0.1% pancreatin (Merck KGaA) in a sterile 0.85% NaCl solution (pH 8) [41]. To evaluate gastric tolerance of spores, suspensions were incubated at 37 °C for 1 h in constant mild shaking. At 0 min (T_0_) and 1 h (T_1_), aliquots (100 μL) were withdrawn, serially diluted, and seeded on TSH plates. To assess survival in the intestinal fluid, suspensions were incubated at 37 °C for 8 h in constant mild shaking. At selected time points (i.e., 0 min (T_0_), 2 h (T_2_), 4 h (T_4_), 6 h (T_6_), and 8 h (T_8_)), aliquots were diluted and streaked on TSH agar plates as for gastric tolerance experiments. Plates were incubated at 37 °C for 48 h. The number of colony-forming units (CFUs) was determined, and the total CFU number per unit dose was calculated.

### 2.4. Mucin Adhesion Assay

Adhesion of spores contained in the formulation to mucins was assessed as previously described [35]. First, 500 µL aliquots of the whole product were added to wells of a 48-well polystyrene microplate containing 600 µL of mucin agar (pH 6.8), constituted by 5% mucins (from porcine stomach type II; Merck KGaA) and 1% bacteriological agar. As negative controls, 500 µL of the formulation was inoculated into wells containing 600 µL of 1% bacteriological agar. Microplates were incubated at 37 °C for 90 min at 30 rpm in aerobic or anaerobic conditions. Anaerobiosis was generated by incubating microplates in the presence of AnaeroGen Compact sachets (Thermo Fisher Scientific). After incubation, supernatants were discarded, and wells were washed twice with sterile PBS to remove non-adherent spores. Then, the solid layers taken from wells were transferred to a sterile tube containing 5 mL of sterile PBS and homogenized for 5–10 min. Aliquots (100 µL) were streaked on TSH agar plates, and the number of adhered spores was quantified using the plate count method.

### 2.5. Spore Germination in the Intestinal Fluid

The content of each vial was aseptically transferred to a sterile tube and centrifuged at 4000× *g* for 10 min at 4 °C. The supernatant was discarded, and spores were activated following two different protocols. The spore pellet was suspended in 5 mL of sterile water, incubated at 70 °C for 30 min to achieve spore activation by high temperature, and then centrifuged at 4000× *g* for 10 min at 4 °C. Alternatively, spores were directly suspended in 5 mL of USP gastric juice (pH 1.5), prepared as above, and incubated at 37 °C for 1 h. At the end of incubation, the suspension was centrifuged at 4000× *g* for 10 min at 4 °C, the supernatant was discarded, and the pellet was washed once with cold sterile water. After centrifugation at 4000× *g* for 10 min at 4 °C, the supernatant was discarded. The pellets of both activated spores were suspended in 5 mL of artificial intestinal fluid, prepared as above, containing 0.5% glucose, 1 mM L-alanine, 1 mM adenosine, and 1 mM inosine as germination inductors [42,43]. A total of 1 ml of the suspension was immediately transferred to a clear cuvette, and germination kinetics were monitored by reading absorbances at OD_600_ every 15 min for up to 4 h by a BioPhotometer (Eppendorf, Hamburg, Germany). A reduction in OD_600_ was expected during spore germination. A 100% germination rate was assumed to correspond to a decrease of OD_600_ ≈ 60% than the initial optical density for intact spores, as previously described for other *Bacillus* species [44].

### 2.6. Quantification of β-Galactosidase

To assess β-galactosidase production, 100 µL aliquots of the formulation were inoculated in 5 mL of LB broth and incubated overnight at 37 °C to allow for spore germination and the growth of vegetative cells. The next day, 100 µL of the culture was diluted in 10 mL of fresh LB broth supplemented with 0.5 mM isopropyl β-D-1-thiogalactopyranoside (IPTG; Merck KGaA) and grown at 37 °C to an optical density at 600 nm (OD_600_) of 0.5. Quantification of the β-galactosidase activity was performed according to the Miller method, as previously described [45]. Briefly, 1 mL of the culture suspension was centrifuged at 8000× *g* at 4 °C for 5 min, and the pellet was suspended in an equal amount of cold Z buffer (60 mM Na_2_HPO_4_ × 7H_2_O, 40 mM NaH_2_PO_4_ × H_2_O, 10 mM KCl, 1 mM MgSO_4_ × 7H_2_O, and 50 mM β-mercaptoethanol (Thermo Fisher Scientific); pH 7). To permeabilize vegetative cells, 20 µL of 0.1% sodium dodecyl sulfate (Merck KGaA) and 40 µL of chloroform were added, and the tubes were mixed by vortexing for 10 s. A total of 100 µL of the samples was diluted in 1 mL of cold Z buffer and incubated with 200 µL of 4 mg/mL orto-nitrofenil-β-galactopyranoside (ONPG; Merck KGaA) at 28 °C until the development of a yellow color. The reaction was stopped by adding 250 µL of 1 M Na_2_CO_3_. β-galactosidase activity was calculated using the Miller formula (Miller units = 1000 × (OD_420_ − (1.75 × OD_550_)/T × V × OD_600_), where OD_420_, OD_550_, and OD_600_ are the optical densities at 420, 550, and 600 nm, respectively, T the reaction time, and V the total volume of culture assayed expressed in milliliters) [46]. *Proteus mirabilis* ATCC 12453 and *Escherichia coli* K12 were included in the assays as negative and positive controls, respectively.

### 2.7. Preparation of Cell Lysates and Culture Supernatants

To prepare cell lysates and culture supernatants of actively replicating cells, 100 µL of the formulation was inoculated in 5 mL of BHIG broth and grown overnight at 37 °C in constant shaking. The next day, 100 µL of culture suspension was inoculated in a fresh BHIG medium and incubated at 37 °C to an OD_600_ of 1.8. Thereafter, cultures were centrifuged at 4000× *g* for 20 min at 4 °C. Supernatants were collected and filtered using 0.22 µm filters, while pellets were suspended in 1 mL of sterile cold PBS. For cell lysis, zirconia beads (ø 0.1 mm) were added to the cell suspensions (ratio 1:1) and homogenized with a BeadBeater homogenizer. Aliquots of cell lysates and culture supernatants were stored at −80 °C until use. The protein content of both lysates and supernatants was determined by the Pierce BCA Protein Assay Kit (Thermo Fisher Scientific), according to the manufacturer’s instructions for the microplate procedure.

### 2.8. Quantification of Catalase (CAT) and Superoxide Dismutase (SOD)

The amount of antioxidant enzymes of CAT and SOD in both lysates and supernatants was evaluated using the colorimetric Catalase Activity Assay kit (Abcam, Cambridge, UK) and Superoxide Dismutase Activity Assay kit (Abcam), respectively, according to the manufacturer’s instructions. The limits of sensitivity were 1 μU/mL and 0.1 U/mL for catalase and superoxide dismutase activities, respectively.

### 2.9. Quantification of Vitamin B_2_, B_8_, B_9_, and B_12_

Vitamin B_2_ (riboflavin) was quantified in culture supernatants using the enzyme-linked immunosorbent assay (ELISA) vitamin B_2_ kit (Cloud-Clone Corp., Katy, TX, USA), according to the manufacturer’s protocol. Vitamins B_8_ (biotin), B_9_ (folic acid), and B_12_ (cobalamin) were quantified in both lysates and supernatants using the SENSISpec ELISA vitamin B_8_/B_9_/B_12_ kits (Gold Standard Diagnostics, Budapest, Hungary), according to the manufacturer’s protocols. The limits of sensitivity were 7.4 ng/mL, 0.5 ng/mL, 2 ng/mL, and 0.3 ng/mL for vitamins B_2_, B_8_, B_9_, and B_12_, respectively. Sterile PBS and BHIG were used as negative controls for cell lysates and culture supernatants, respectively.

### 2.10. Quantification of Short-Chain Fatty Acids

Quantification of acetic, propionic, and butyric acids in culture supernatants was performed using the improved method of high-performance liquid chromatography coupled with tandem mass spectrometry (HPLC-MS/MS) specific for short-chain fatty acids (SCFAs), as previously reported [47]. Briefly, supernatants underwent liquid–liquid extraction and derivatization before HPLC-MS-MS analysis. An ultra-HPLC Infinity II system (Agilent, Santa Clara, CA, USA) coupled to a QTRAP 6500 + LC-MS-MS (Sciex, Concord, ON, Canada) was used. Chromatographic separation was achieved by using a Gemini C18 HPLC column (Phenomenex, Torrance, CA, USA). Quantification of SCFAs was performed using calibration curves obtained by serially diluting stock standard solutions to concentration ranges of 1.95–1000 ng/mL for acetic acid and 0.39–200 ng/mL for propionic and butyric acids. The amount of acetic, propionic, and butyric acids detected in sterile BHIG was subtracted from the values obtained for culture supernatants.

### 2.11. Evaluation of D-Lactic Acid Production

Culture supernatants were deproteinized by precipitating proteins with 10% 6 N tricarboxylic acid for 16 h at 4 °C [48]. After incubation, deproteinized supernatants were collected, and the presence of D-lactate was tested by using the colorimetric D-Lactate Assay kit (Abcam), according to the manufacturer’s instructions. Deproteinized BHIG and supernatant obtained from *Limosilactobacillus reuteri* DSM 17938 were used as negative and positive controls, respectively.

### 2.12. Statistical Analysis

Experiments were repeated three times on separate days with two technical replicates each. Data were expressed as mean ± standard deviation. A two-tailed Student’s *t*-test for paired data was applied to assess statistical significance in the gastric tolerance and mucin adhesion experiments. To assess spore tolerance to intestinal conditions, the one-way ANOVA for repeated measures was used, followed by the Tukey test for multiple comparisons. A two-sided *p*-value of <0.05 was considered significant.

## 3. Results and Discussion

### 3.1. Total Count

The product content was preliminarily evaluated to verify the quality of the formulation in terms of the number of spores and vegetative cells. The total cell count, including both vegetative cells and spores, resulted in 2.15 × 10^9^ ± 2.68 × 10^8^ CFU/unit dose. Spores alone were quantified as 1.66 × 10^9^ ± 2.63 × 10^8^ CFU/unit dose, which is compliant with the amount of spores claimed on the product label (2.00 × 10^9^ CFU/unit dose) (*p* > 0.05). The numbers of spores and total cells were not significantly different, thus indicating that the product is entirely constituted by spores.

### 3.2. Behavior in Simulated Gastrointestinal Conditions: Survival, Adhesion, and Germination

To investigate the behavior of *A. clausii* spores in the gastrointestinal tract, the spores contained in the whole formulation were incubated in artificial fluids simulating gastric and intestinal conditions and tested for adhesion to mucins and germination.

Once orally administered, probiotics must survive the passage through the stomach, where a harsh acidic environment is present. Spores are naturally resistant to such conditions, and the maintenance of their viability through the stomach is considered an indicator of good quality of spores [49]. *A. clausii* spores tolerated the very low pH and the presence of pepsin in the simulated gastric fluid well, not showing any significant alteration in microbial viability at 1 h after inoculation (T_0_ = 1.48 × 10^9^ ± 5.80 × 10^7^ CFU/unit dose; T_1_ = 1.52 × 10^9^ ± 1.86 × 10^8^ CFU/unit dose; *p* > 0.05). Although this result was not surprising due to the intrinsic properties of spores, these findings indicate that *A. clausii* spores could persist unaffected in harsh environments, including the human stomach.

**Figure 1:**
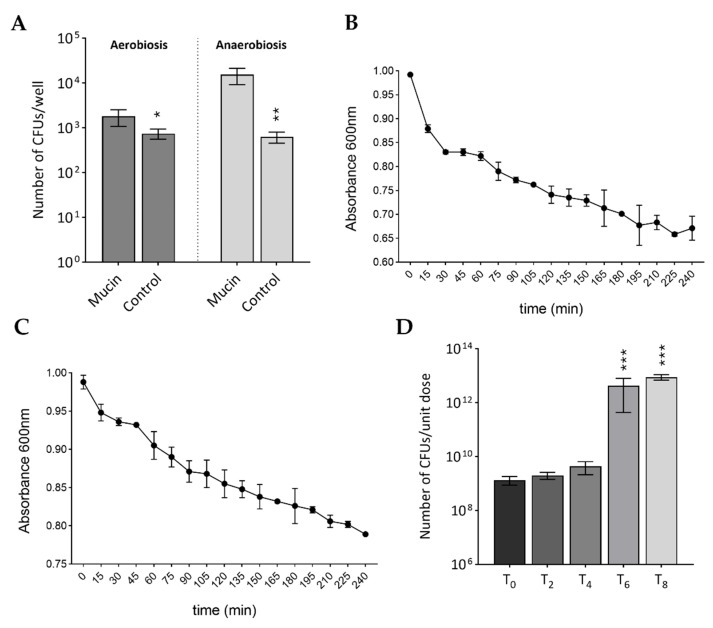
(**A**) Ability of *A. clausii* spores to adhere to mucins in aerobic and anaerobic conditions expressed as number of CFUs/well. (**B**) Germination kinetics of *A. clausii* spores in artificial intestinal fluid supplemented with germination inductors after heat activation. (**C**) Germination kinetics of *A. clausii* spores in artificial intestinal fluid supplemented with germination inductors after pH activation. (**D**) Viability of *A. clausii* spores in simulated intestinal fluid expressed as number of CFUs/unit dose. * *p* < 0.05; ** *p* < 0.01; *** *p* < 0.001.

Adherence of probiotics to the intestinal mucosa is also considered a key feature of bacteria with probiotic claims, since adhesion to mucus limits washout and favors transient colonization of the gut, which is required for probiotics to exert their beneficial effects and enhance resistance to enteropathogens [50,51,52]. For this reason, we tested the ability of *A. clausii* spores to adhere to mucins either in the presence or absence of oxygen. As shown in Figure 1A, the number of CFUs recovered from mucins was significantly higher than that of the negative control in both aerobic (*p* < 0.05) and anaerobic conditions (*p* < 0.01), thus indicating that *A. clausii* spores are able to adhere to mucins *in vitro*. Although vegetative *A. clausii* strains NR, OC, SIN, and T isolated from Enterogermina^®^ were shown to adhere to mucins in a previous *in vitro* study [35], to the best of our knowledge, this is the first report evidencing the mucin-adhesive ability of their spores. Our findings are in line with the demonstrated ability to bind mucins of spores of an ileal isolate of *A. clausii* [53].

To temporarily colonize the host’s gut and impart benefits, probiotics need to be vital, active, and able to multiply in intestinal alkaline conditions [8]. Spore germination in the gut is the first step for spore-based formulations to exert the claimed beneficial effect on the host. Although different behaviors are expected upon administration *in vivo* due to the presence of a plethora of nutrients and natural germination inductors in the intestine, *in vitro* germination in conditions mimicking those found in the gut represents the only chance to monitor spore germination of probiotic spore-forming bacilli. Spores contained in the whole formulation successfully germinated, with a rate of 55.9 ± 0.6% after heat activation (Figure 1B) and 33.1 ± 1.8% after pH activation (Figure 1C). The steepest slopes were observed in the very first 15 min after both heat and pH spore activation, in which 18.8 ± 1.2% and 6.7 ± 3.3% of the total spores germinated, respectively. Thus, although high temperature resulted in more proficient activation of *A. clausii* spores and induced germination in *in vitro* conditions, a low pH was sufficient to sustain the activation and germination of a considerable number of spores.

Bile resistance and multiplication in the presence of bile are also considered desirable properties that are used to select potential probiotic strains. As shown in Figure 1D, the number of CFUs of *A. clausii* contained in the pancreatin–bile salt solution was stable for up to 4 h and then significantly increased (*p* < 0.001 compared to T_0_), thus indicating that *A. clausii* spores are able to germinate and vegetative cells are able to proliferate in the simulated intestinal fluid. The ability of *A. clausii* spores to survive and multiply in artificial intestinal fluid for a prolonged duration and persist in the human gut has been evidenced by multiple studies and is potentially attributable to the high tolerance of *A. clausii* spores to harsh intestinal conditions [12,35,54,55,56,57,58]. By analyzing the ENTpro metagenome obtained from the whole Enterogermina^®^ formulation, five genes encoding proteins potentially implicated in bile tolerance were identified, two of which belong to the ornithine decarboxylases and three to the sodium bile acid symporter family [59]. The presence of these genes could justify the bile resistance exhibited by the mixture of the four *A. clausii* strains constituting the product.

Altogether, the mucin adhesion properties and the behavior of the whole product in simulated gastrointestinal conditions suggest that these properties are responsible for the extended resilience of *A. clausii* in the intestine of human volunteers who consumed the formulation [12].

### 3.3. Production of Beneficial Enzymes

Some probiotics demonstrated efficacy in the management of lactose intolerance [60]. Human digestion of lactose is the result of the activity of lactase (formerly β-galactosidase), an enzyme that hydrolyses lactose to glucose and galactose, which can be absorbed in the small intestine. A lack of β-galactosidase production/activity results in lactose intolerance, but sufficient hydrolysis of lactose in the small intestine may prevent symptoms of lactose intolerance. Therefore, probiotics with the ability to produce lactose-degrading enzymes gained interest as potential compensation for lactase deficiency [6,35,61]. In general, *Streptococcus thermophilus* and *Lactobacillus delbrueckii* spp. *bulgaricus* are known to be effective against lactose intolerance [61]. In a previous report, we also evidenced that *A. clausii* strains are able to produce β-galactosidase [35]. As shown in Table 1, the mixture of *A. clausii* strains after growing the whole formulation in a liquid medium was able to synthesize substantial amounts of β-galactosidase (61.76 ± 13.49 Miller units), thus suggesting that its administration could be helpful in reducing symptoms of lactose intolerance *in vivo*.

The ability of probiotics to exert health-promoting effects through the production of enzymes with antioxidant activity was also widely studied in recent decades [36,62]. Oxidative stress, a condition in which the redox balance in the cell is altered, results in DNA hydroxylation, protein denaturation, lipid peroxidation, and apoptosis, ultimately compromising cell viability. The production of enzymes with antioxidant activity, such as catalase and superoxide dismutase, was proposed as one of the mechanisms that probiotics exert to modulate antioxidation [62,63,64]. CAT participates in cellular antioxidant defense by decomposing hydrogen peroxide, thereby preventing the generation of hydroxyl radicals by the Fenton reaction, while SOD catalyzes the breakdown of superoxide into hydrogen peroxide and is a central regulator of the levels of reactive oxygen species. Such enzymes potentially offer a novel approach to the management of bowel diseases characterized by a substantial production of reactive oxygen species in the gut [65,66,67,68]. In this study, we found that the whole formulation produced and secreted important levels of SOD (lysate: 1.00 × 10^3^ ± 1.02 × 10^2^ ng/mL; supernatant: 6.62 × 10^2^ ± 1.71 × 10^2^ ng/mL) and CAT (lysate: 0.02 ± 0.00 ng/mL; supernatant: 0.18 ± 0.08 ng/mL) to a lesser extent (Table 1). Overall, these findings suggest that *A. clausii* may contribute to the prevention or treatment of oxidative stress injury, considering that antioxidant mechanisms other than SOD and CAT may also be involved in this defense.

### 3.4. Production of Vitamins and SCFAs

One of the benefits conveyed by probiotics is the production of beneficial metabolites, such as vitamins and SCFAs. Humans are unable to synthesize many vitamins and obtain them daily from exogenous sources, such as food, dietary supplements, and food additives [69]. B vitamins, such as vitamins B_2_ (riboflavin), B_8_ (biotin), B_9_ (folic acid), and B_12_ (cobalamin), play a central role in cell metabolism and the maintenance of many physiological functions in humans [70]. In particular, riboflavin is required for carbohydrate, protein, and fatty acid metabolism and is the precursor of other B vitamins, while biotin is an essential cofactor in carboxylation/decarboxylation reactions, contributes to immune functions, and maintains intestinal mucosa integrity [71,72,73]. Folic acid and cobalamin are involved in nucleic acid synthesis and hematopoiesis [70]. Only few reports demonstrating the production of B vitamins by *A. clausii* are available in the literature [35,36,74,75,76,77]. In this study, we quantified the amount of vitamin B_2_ secreted by the mixture of the four *A. clausii* strains constituting the whole formulation. As shown in Table 1, the *A. clausii* mixture secreted 22.86 ± 1.61 ng/mL of riboflavin, an amount that is very similar to that produced by single *A. clausii* strains [35]. Then, we investigated for the first time the ability of the whole formulation to produce/secrete vitamins B_8_, B_9_, and B_12_. These vitamins were detected in both cell lysates and culture supernatants in comparable amounts: B_8_ (lysate: 5.89 ± 0.03 ng/mL; supernatant: 3.93 ± 0.01 ng/mL), B_9_ (lysate: 3.11 ± 0.32 ng/mL; supernatant: 2.01 ± 0.12 ng/mL), and B_12_ (lysate: 1.48 ± 0.15 ng/mL; supernatant: 1.17 ± 0.64 ng/mL). These results are in line with data showing that the *A. clausii* B106 genome contains the genes required for the synthesis of vitamins B_8_ and B_9_ [78]. In addition, the microbial secretion of folates and cobalamin was previously reported in probiotic strains, including *A. clausii* [79].

SCFAs, the largest group of metabolic products obtained from the microbial fermentation of carbohydrates and dietary fibers by the gut microbiota [80,81], orchestrate several physiological functions to ensure host health [82]. Since the impairment of SCFA levels correlates with the onset of different pathological conditions, including inflammatory bowel disease [83], restoring appropriate intestinal concentrations of SCFAs in SCFA-deficient patients may allow clinicians to ameliorate disease-associated symptoms. Several studies demonstrated the ability of some probiotics to produce and actively release acetate, propionate, and butyrate in different amounts and in a species- or strain-specific manner [5,47]. In the current study, we found that the mixture of *A. clausii* strains constituting the probiotic formulation secreted the three main SCFAs in culture supernatants, with high amounts of acetic acid (586.67 ± 39.37 ng/mL) and, to a lesser extent, propionic (0.79 ± 0.14 ng/mL) and butyric acids (2.85 ± 0.46 ng/mL) (Table 1).

Once administered, the *A. clausii* mixture could contribute to the overall production of group B vitamins and SCFAs in the human gut, potentially ameliorating intestinal dysvitaminosis and SCFA deficiency.

### 3.5. Production of D-Lactate

As recent studies indicated that an excess of D-lactic acid production by certain probiotic bacteria may cause symptoms such as brain fog, abdominal pain, and bloating [84,85], the administration of D-lactate-producing strains should be carefully considered, especially in patients at risk of developing D-lactic acidosis, such as those with short bowel syndrome, newborns, and neonates [86]. The accumulation of serum D-lactate can also result in neurotoxic effects, such as delirium, ataxia, and slurred speech [87]. As such, the finding that the *A. clausii* mixture could not synthesize D-lactic acid at all during growth (Table 1) mitigates any risk of product administration in those subjects potentially prone to developing D-lactic acidosis.

## 4. Conclusions

The current analysis supports the beneficial effects of Enterogermina^®^ formulation containing spores of *A. clausii* on intestinal health. Herein, we showed that *A. clausii* spores excellently tolerated gastrointestinal conditions, germinated and multiplied in the presence of bile salts, and adhered to mucins. The ability to produce beneficial enzymes, group B vitamins, and SCFAs may be considered an additional value that ameliorates human health and further strengthens the use of *A. clausii* in the probiotic field. However, further research is likely to identify additional benefits across other therapeutic areas and establish additional modes of action responsible for the therapeutic benefits of *A. clausii*.

## Figures and Tables

**Table 1:** Summary of the molecules produced by the formulation of four strains of *A. clausii*.

**Production of Group B Vitamins (ng/mL)**		**Mean ± SD**
Vitamin B_2_	Supernatant	22.86 ± 1.61
Vitamin B_8_	Cell lysate	5.89 ± 0.03
	Supernatant	3.93 ± 0.01
Vitamin B_9_	Cell lysate	3.11 ± 0.32
	Supernatant	2.01 ± 0.12
Vitamin B_12_	Cell lysate	1.48 ± 0.15
	Supernatant	1.17 ± 0.64
**Production of enzymes with antioxidant activity (ng/mL)**		
Superoxide dismutase	Cell lysate	1000.82 ± 102.15
	Supernatant	661.95 ± 171.47
Catalase	Cell lysate	0.02 ± 0.00
	Supernatant	0.18 ± 0.08
**Production of β-galactosidase** **(Miller units)**		
β-galactosidase		61.76 ± 13.49
**Secretion of short-chain fatty acids (ng/mL)**		
Acetic acid	Supernatant	586.67 ± 39.37
Propionic acid	Supernatant	0.79 ± 0.14
Butyric acid	Supernatant	2.85 ± 0.46
**Secretion of D-lactic acid**		
D-lactate	Supernatant	Not produced

## Data Availability

Datasets generated during the current study will be made available from the corresponding author upon reasonable request.
